# PERSONALITY TRAITS ARE ASSOCIATED WITH THE TYPES OF SOCIAL SUPPORT/STRAIN: A LATENT PROFILE ANALYSIS

**DOI:** 10.1093/geroni/igae098.2429

**Published:** 2024-12-31

**Authors:** Meredith Willard, Jacob Alderson, Nicholas Turiano

**Affiliations:** West Virginia University, Morgantown, West Virginia, United States; West Virginia University, Morgantown, West Virginia, United States; West Virginia University, Morgantown, West Virginia, United States

## Abstract

Research suggests that social connections are important for health and longevity (Cohen, 1985). High levels of social support is a significant predictor of improved well-being and health (White et al., 2009). However, it is important to acknowledge the role social strain can have on health, as well as key predictors of social network functioning. Thus, the current study utilized data from the Midlife Development in the US Study (MIDUS) to extract classes of social support/strain using a latent profile analysis (LPA), and use the Big 5 personality traits to predict class membership (N = 6,178; Mean age = 46.8). Social support/strain was separately computed for each network source (i.e., family, friend, and spouse) using 4-6 items. Iterations of different LPA classes suggested a 4-class solution (lowest AIC/BIC;.83 entropy). Class 1 (high spouse strain and lowest levels of support); Class 2 highest support and lowest strain levels);. Class 3 (highest level of spouse support and low levels from friends and family); Class 4 (average levels of support and strain across all sources). Those high in Conscientiousness and Agreeableness were more likely to belong in the “optimal” class of high support & low strain while those high in Openness and Neuroticism were more likely to belong in the “average” class (all p’s <.05). Findings underscore how personality traits are involved in the exposure to varying levels of support/strain individuals are exposed to. Understanding these processes is key to understanding how social networks impact aging outcomes.

